# Exploration of the role of gene mutations in myelodysplastic syndromes through a sequencing design involving a small number of target genes

**DOI:** 10.1038/srep43113

**Published:** 2017-02-21

**Authors:** Feng Xu, Ling-Yun Wu, Qi He, Dong Wu, Zheng Zhang, Lu-Xi Song, You-Shan Zhao, Ji-Ying Su, Li-Yu Zhou, Juan Guo, Chun-Kang Chang, Xiao Li

**Affiliations:** 1 Department of Hematology, Shanghai Jiao Tong University Affiliated Sixth People’s Hospital, Shanghai, China.

## Abstract

Novel sequencing designs are necessary to supplement the recognized knowledge of myelodysplastic syndrome (MDS)-related genomic alterations. In this study, we sequenced 28 target genes in 320 Chinese MDS patients but obtained 77.2% of recall factors and 82.8% of genetic abnormalities (including karyotype abnormalities). In addition to known relationships among mutations, some specific chromosomal abnormalities were found to link to specific gene mutations. Trisomy 8 tended to be linked to U2AF1 and ZRSR2 mutations, and 20q- exhibited higher SRSF2/WT1 and U2AF1 mutation frequency. Chromosome 7 involvement accounted for up to 50% of RUNX1 mutations and 37.5% of SETBP1 mutations. Patients carrying a complex karyotype were prone to present TP53 mutations (36.1%). However, individuals with normal karyotypes rarely possessed mutations in the TP53, RUNX1 and U2AF1. Moreover, DNMT3A, TP53, SRSF2, STAG2, ROBO1/2 and WT1 predicted poor survival and high AML transformation. By integrating these predictors into international prognostic scoring system (IPSS) or revised IPSS, we built a set of mutation-based prognostic risk models. These models could layer different degrees of risk in patients more satisfactorily. In summary, this sequencing design was able to detect a number of gene mutations and could be used to stratify patients with varied prognostic risk.

Myelodysplastic syndromes (MDSs) are a heterogeneous group of myeloid neoplasms characterized by varying degrees of cytopenia, dysplasia and a high risk of progression to acute myeloid leukemia (AML)^1^. Due to the conspicuous clinical and biological heterogeneity of MDS, an optimized choice of treatment based on an accurate diagnosis and risk stratification in individual patients is central to the therapeutic strategy^2^. In addition to IPSS^3^ involving three parameters (conventional cytogenetics, peripheral blood counts and the bone marrow blast ratio), major mutational targets that were identified in previous studies have been successfully used for this purpose^4^,^5^,^6^. These mutations have mainly pointed to newly identified pathways, including epigenetic regulators and the RNA-splicing machinery, although previously defined AML-related signal-transducing molecules and transcription factors are also involved^7^,^8^,^9^,^10^. When more than 100 target genes were sequenced, at least one mutation could be found in approximately 80–90% of subjects (including some with MDS/MPN and MDS-AML)^7^,^8^. The distribution of MDS-related gene mutations is known to be highly heterogeneous, except for several mutations with a frequency over 10%^7^,^8^. Some low-frequency mutations point to characteristics of marrow proliferation (JAK2/CALR/MPL) and de novo AML (N-RAS or FLT3)^7^,^8^. In the present study, we focused on a simple study design involving a combination of fewer than 30 target genes to screen our MDS patients (excluding those with MDS/MPN or MDS-AML) to determine recall factor levels as well as whether any novel insights could be obtained and if this combination is helpful for further stratifying different degrees of risk in patients based on revised international prognostic scoring system (IPSS-R)^11^. This small combination of target genes was obtained from both previous studies by others and our own published findings^12^.

## Methods and Materials

### Patients and samples

Samples were obtained from 320 MDS patients without characteristics of MPN or transformed AML at our center between January 2009 and August 2014. MDS was diagnosed in accordance with the minimum diagnostic criteria (Vienna, 2006)^13^. The patients were subclassified into the refractory cytopenia with unilineage dysplasia (RCUD), refractory anemia with ringed sideroblasts (RAS), refractory cytopenias with multilineage dysplasia or with ringed sideroblasts (RCMD-RS), refractory anemia with excess blasts (RAEB-1), RAEB-2, and MDS with sole 5q- groups according to the WHO 2008 classification^14^. Their prognosis was evaluated based on the IPSS-R^11^. Bone marrow (BM) samples were obtained via aspiration from all of the patients when the diagnosis was made, prior to receiving any treatment with chemotherapy or hypomethylation agents, and mononuclear cells were subsequently collected via density gradient centrifugation. The ethics committee of the Sixth Hospital affiliated with Shanghai Jiao Tong University approved this research. All subjects provided informed consent in accordance with the Declaration of Helsinki. The methods were carried out in accordance with the approved guidelines.

### Genomic DNA preparation

Genomic DNA (gDNA) was extracted from bone marrow mononuclear cells or matched oral mucosa epithelia from the MDS patients. The purity (OD260/280 > 1.8) and concentration (50 ng per μl) of the gDNA met the sequencing requirements.

### Targeted sequencing

The 28 selected mutational gene targets related to MDS were examined for mutations in all 320 cases from the cohort through MiSeq sequencing (Illumina, San Diego, CA, USA). To identify mutations in the highlighted genes, we designed PCR primers using the primer XL pipeline. A total of 560 oligonucleotide pairs were produced, encompassing all of the coding sequences (CDS) and most of the UTRs of the 28 genes. The amplification reactions were conducted using an ABI 2720 Thermal Cycler with the following cycling conditions: 95 °C for 2 min; 11 cycles of 94 °C for 20 s, 63 °C per cycle for 40 s and 72 °C for 1 min; 24 cycles of 94 °C for 20 s, 65 °C for 30 s and 72 °C for 1 min; and 72 °C for 2 min. The PCR products were used to generate a library for further detection, and the DNA adapter-ligated and adapter-indexed fragments from 10 libraries were then pooled and hybridized. After hybridization of the sequencing primer, base incorporation was performed in a single lane using a MiSeq Benchtop Sequencer following the manufacturer’s standard cluster generation and sequencing protocols, for 250 cycles of sequencing per read to generate paired-end reads including 250 bps at each end and 8 bps of the index tag.

### Mutation calling

Sequence data were analyzed through our established pipeline to detect possible somatic mutations. All of the sequencing reads were aligned to the human reference genome (hg19) using BWA version 0.5.8 with default parameters. After all of the duplicated reads and low-quality reads and bases were removed, the allele variable frequencies (AVFs) of single-nucleotide variants (SNVs) and indels were calculated at each genomic position by enumerating the relevant reads using SAMtools. All of the variants showing an AVF >10% were extracted and annotated with ANNOVAR for further consideration only if they were found in >5 positive reads among >10 total reads. All of the synonymous variants and ambiguous (unknown) candidates were discarded. In addition, known SNVs with a frequency greater than 0.01 in the 1000 Genomes databases were removed, though SNVs that were found in the COSMIC database were rescued as somatic mutations.

### Statistical analysis

Statistical analyses were conducted using SPSS software version 18.0. The association of the mutations with clinical characteristics was analyzed via the χ2 test. Comparisons of two independent samples were assessed using the two-tailed Student’s t-test. Fisher’s exact test was applied to determine the co-occurrence of highly recurrent genes. Overall survival (OS) was defined as the time from the date of diagnosis to death or survival at the last follow-up (censored). AML-free survival was calculated from diagnosis to AML progression at the last follow-up (censored). Kaplan-Meier analysis was used to evaluate the time to survival and time to progression. Univariate analyses were performed by using COX proportional hazards regression analyses, among the 308 patients with available survival data (12 patients are lost to follow-up) to assess the impact of age, gender, IPSS-R and gene mutations, as variables, on OS and AML-free survival and to screen the main prognostic factors. The mutation index obtained using the weighted coefficients (log HR) and *P* values from COX analyses of each prognostic factor was used to build the prognostic models. For survival model, the mutations with univariate COX *P* < 0.05 (corresponding log HR range 1.59–2.34, mean 2.08) were scored 2 and the mutations with univariate COX *P* > 0.05 (corresponding log HR range 0.38–1.77, mean 1.16) were scored 1. For AML transformation model, in view of improving the integration of model with IPSS or IPSS-R and reduce bias caused by limited cases with AML transformation, the mutations with univariate COX *P* < 0.05 (corresponding log HR range 1.83–4.09, mean 3.6) were also scored 2 and the mutations with univariate COX *P* > 0.05 (corresponding log HR range 0.29–2.3, mean 1.4) were scored 1. All *P* values were based on 2-sided tests, and *P* values less than 0.05 were considered statistically significant.

## Results

### Patient characteristics

**Figure 1:**
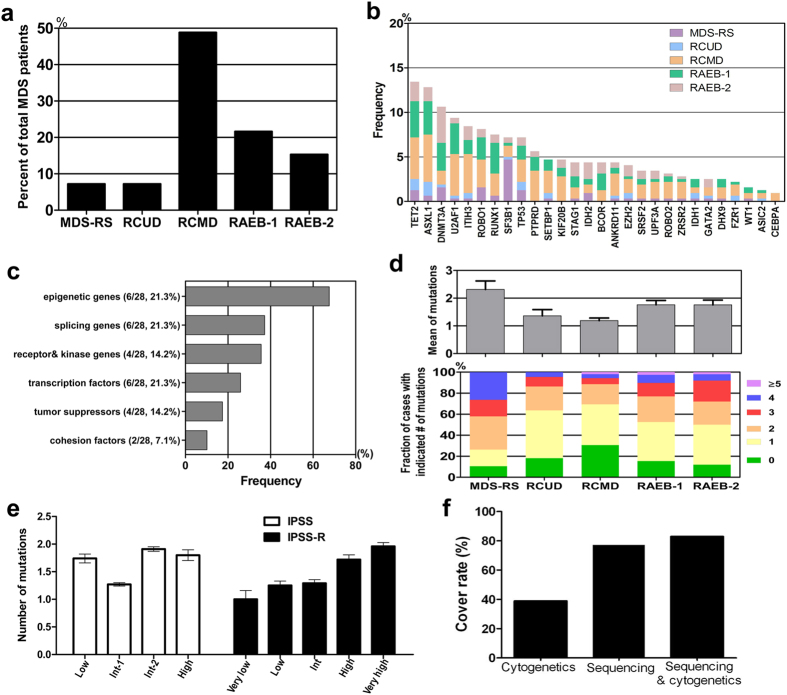
Distribution of gene mutations in 320 MDS patients. (**a**) Percentage of each MDS subtype in total MDS patients is shown. All patients with RS are grouped as MDS-RS. (**b**) Frequen*cy of* mutatio**ns** in 28 significantly mutated genes in 320 cas**es** with different WHO subtypes, which are shown in the indicated colors. (**c**) Frequency of gene mutations involved in common functional pathways. 28 mutated genes are grouped into six functional pathways including epigenetic modifiers, splicers, transcription factors, receptor kinases, tumor suppressors and cohesion factors. (**d**) Number of gene mutations detected in different MDS subtypes. MDS with RAEB-1 or RAEB-2 show more number of mutations and higher percentage of cases with over 3 mutations than those with RCUD or RCMD Fig. 1d. Notably, besides SF3B1, the patients with MDS-RS also have more number of mutations compared with those with RCUD or RCMD. (**e**) Number of gene mutations according to different IPSS or IPSS (R) scores.The mean number of mutations seems to show a correlation with IPSS-R risk categories. (**f**) Fraction of patients with at least 1 mutation identified based on cytogenetics, targeted gene sequencing, or sequencing combined with bone marrow cytogenetics. In total, one genetic abnormality at least is identified through combination analysis of cytogenetics with sequencing in 265 of 320 cases.

In total, 320 patients were enrolled in this study, including 178 men and 142 women with a median age of 57 years. Their WHO classification, karyotype status and IPSS(R) risk scores are detailed in Table 1. In view of the specific role of SF3B1 in ringed sideroblasts (RS), all patients with RS including 18 cases with RAS, 2 cases with RCMD-RS, 2 cases with RAEB-1-RS and 1 case with RAEB-2-RS were grouped as MDS-RS (Fig. 1a).

### Candidate gene panel for targeted sequencing

A designated panel of 28 genes was selected to generate a custom sequencing library. Some recognized MPN-related genes (JAK2/CALR/MPL) and de novo AML-related genes (e.g., NPM1, N/K-RAS, FLT3) were excluded. These genes were selected according to the following criteria: (i) mutated gene with high frequency recommended by MDS NCCN guideline^15^, with frequency of occurrence over 5% including ASXL1 (15–25%), DNMT3A (12–18%), EZH2 (5–10%), RUNX1 (10–15%), SF3B1 (20–30%), SRSF2 (10–15%), STAG2 (5–10%), TET2 (20–25%), TP53 (8–12%), U2AF1 (8–12%) and ZRSR2 (5–10%), recurrent gene mutations in other MDS/MPN such as SETBP1 (5–10%) and BCOR (5–10%), or gene mutations associated with AML transformation in MDS including CEBPA (<5%), GATA2 (<5%), IDH1 (<5%), IDH2 (<5%) and WT1 (<5%); and (ii) gene mutations that were newly observed in the MDS disease progression stage or recurrent mutations identified through paired whole-exome or targeted sequencing of patients in our previous study^12^, including DHX9 (9.6%), ROBO1 (7.2%), ANKRD11 (6.2%), ROBO2 (5.3%), PTPRD (5.1%), ITIH3 (4.3%), KIF20B (4.3%), UPF3A (2.9%), FZR1 (2.4%), and ASIC2 (2.0%). These newly identified mutations were related to the development of cancer and most of them were present in the COSMIC database.

### Targeted sequencing

The mean depth of the targeted sequencing analysis for the 28 genes was 250-fold (32–4221 reads) across the entire cohort (n = 320). A total of 481 single-nucleotide variants and insertions/deletions (indels) in the 28 genes were called as high-probability somatic changes.

### Frequency and spectrum of gene mutations

The six most frequently mutated genes were TET2 (43 cases, 13.4%); ASXL1 (41 cases, 12.8%); DNMT3A (34 cases, 10.6%); U2AF1 (30 cases, 9.4%); ITIH3 (27 cases, 8.4%) and ROBO1 (26 cases, 8.1%). The genes harboring less common mutations (5–8%) included RUNX1 (7.5%), SF3B1 (7.2%), TP53 (7.2%) and PTPRD (5.6%). Most of the significantly mutated genes were previously well documented either in MDS or other myeloid malignancies, although some were newly identified or re-confirmed as recurrently mutated genes in our analysis, such as ROBO1/2, IHIT3, KIF20B, PTPRD, ANKRD11 and DHX9 (Fig. 1b). Compared with foreign data^7^,^8^, Chinese MDS patients presented a very low SRSF2 mutation ratio (3.4% vs. over 10%). 28 mutated genes were grouped into six functional pathways including epigenetic modifiers (6/28), splicers (6/28), transcription factors (6/28), receptor kinases (4/28), tumor suppressors (4/28) and cohesion factors (2/28), which were hypothesized to characterize MDS pathogenesis (Fig. 1c). Among these genes, the most frequent targets were epigenetic modifiers, in which mutations were observed in as many as 67.6% of cases, followed by genes associated with RNA splicing (37.2%), receptor kinases (35.6%), transcription 25.9%, tumor suppression (17.4%) and cohesion (10.1%) (Fig. 1c). When the data were analyzed according to WHO classification, the mean number of mutations appeared to be similar between RCUD and RCMD (1.36 vs. 1.19) and between RAEB1 and RAEB2 (1.74 vs. 1.69), except for MDS-RS (mean of 2.39 mutations for 23 patients with 2 RAS and 18 RCMD-RS or 2 RAEB-1-RS and 1 RAEB-2-RS) (Fig. 1d). MDS with RAEB-1 or RAEB-2 showed higher percentage of cases with over 3 mutations than those with RCUD or RCMD (Fig. 1d). SF3B1 mutations were frequently confirmed in MDS-RS (15/23, 65.2%). When the data were analyzed according to IPSS, the mean number of mutations was 1.74, 1.27, 1.91, and 1.80 in the low risk, intermediate 1, intermediate 2 and high-risk groups, whereas the corresponding value was 1.0, 1.25, 1.29, 1.72 and 1.96 respectively according to the revised IPSS (Fig. 1e). In total, 247 of the 320 patients (77.2%) harbored at least one mutation, among whom 142 (57.5%) exhibited a normal karyotype. Among 73 cases without detectable mutations, 18 displayed cytogenetic abnormalities. When the molecular/cytogenetic data were combined, we found at least one genetic abnormality in 265 of 320 cases (82.8%) (Fig. 1f). Taken together, these data provide a landscape of genetic alterations in MDS.

### Cooperative or exclusive relationships among distinct gene categories

**Figure 2:**
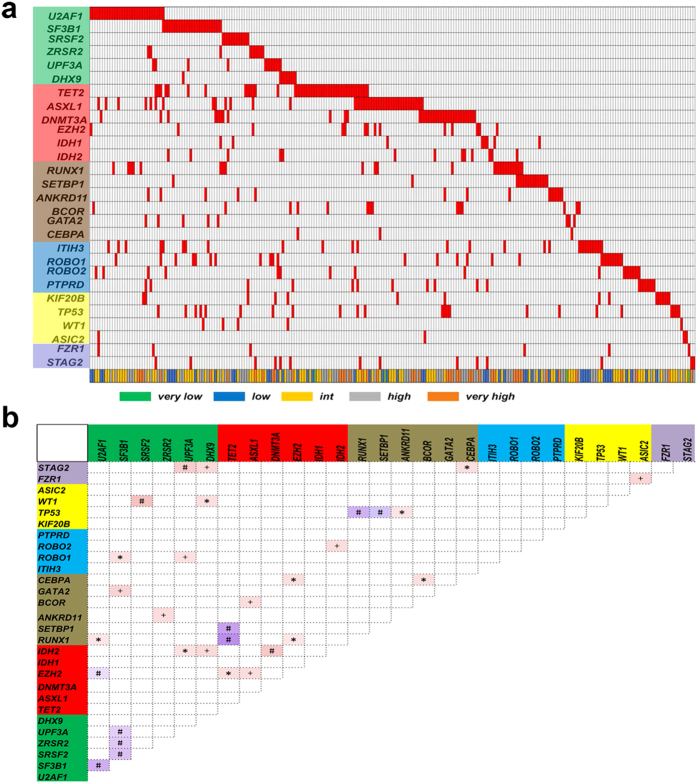
Cooperative or exclusive relationships between varied gene mutation catalogs. (**a**) General illustration of exclusive relationships in 320 MDS cases. Red bar in the X axis indicates the presence of the gene mutation in the respective sample. Six gene function categories in the Y axis includes splicers (green background), epigenetic modifiers (red), transcription factors (brown), receptor kinases (blue), tumor suppressors (yellow) and cohesion factors (purple). Different risk categories are shown in different colors according to the IPSS-R. Splicing gene mutations show a significant exclusive relationship with each other. (**b**) The correlations and coefficients between gene mutations are indicated by a color gradient. Red indicates a cooperative relationship, and purple indicates an exclusive relationship. *q < 0.05; ^+^q < 0.01; ^#^q < 0.001.

The landscape of somatic alterations identified in our analysis revealed several known and unknown associations of gene mutations and indicated mutual exclusion of other genetic alterations (Fig. 2a). In detail, Fig. 2b shows q value < 0.001 for the cooperative relationships between the following three gene mutation pairs: the cohesin family gene STAG2 and the spliceosome gene UPF3A; WT1 and SRSF2; and IDH2 and DNMT3A. A q value < 0.01 for the cooperative relationship was observed between STAG2 and DHX9; FZR1 and ASIC2; ROBO2 and IDH2; ROBO1 and UPF3A; GATA2 and SF3B1; BCOR and ASXL1; ANKRD11 and ZRSR2; IDH2 and DHX9; and EZH2 and ASXL1. Thereafter, the cooperative relationships were relatively weak (q < 0.05) including those between TP53 and ANKRD11; ROBO1 and SRSF2; and RUNX1 and U2AF1. On the other hand, mutually exclusive models could exist between genes in the same functional categories. For example, TP53 mutations (which have been recognized as a predictor of poor prognosis) did not coexist with the reported disease progression-related RUNX1/SETBP1 mutation. Mutual exclusion of gene mutations was also observed among different splicing modifiers. Interestingly, the so-called “good” TET2 mutation showed mutual exclusion in relation to RUNX1 and SETBP1.

### Cooperative relationships among distinct karyotypes and gene categories

**Figure 3:**
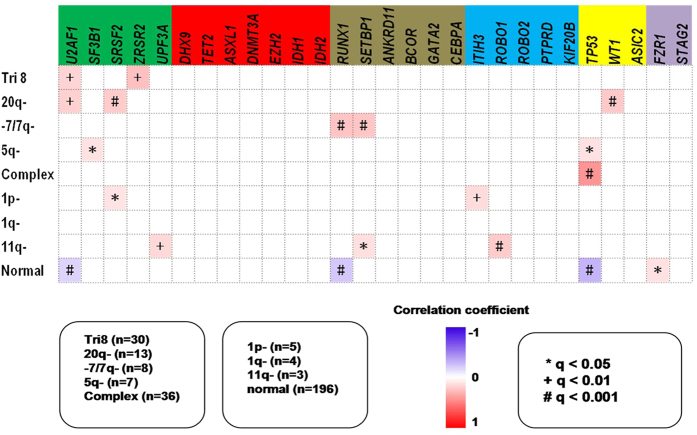
Specific relationships between gene mutations and chromosomal abnormalities. The correlations and coefficients between gene mutations and chromosomal abnormalities are indicated by a color gradient. Red indicates a cooperative relationship, and purple indicates an exclusive relationship. *q < 0.05; ^+^q < 0.01; ^#^q < 0.001.

We analyzed the cooperative or exclusive relationships among distinct karyotypes and gene mutations (Fig. 3). Interestingly, most of the recurrent abnormal karyotypes presented some relationship with specific gene mutations. Trisomy 8 frequently coexisted with U2AF1 and ZRSR2 mutations; 20q deletion coexisted with SRSF2, WT1 and U2AF1 mutations; −7/7q- frequently coexisted with RUNX1 and SETBP1 mutations (at frequencies reaching 50% and 37.5%, respectively) and 5q- coexisted with SF3b1 and TP53 mutations (Supplemental Table 1). Notably, the patients with complex karyotypes exhibited a close correlation with TP53 mutations (36.1% recurrence). “Very good” 11q- presented a positive relationship with poor ROBO1 mutations, although 11q- was found in only three patients (two of the three cases harbored only 11q-, while the third harbored 11q- plus 12p-). The analysis of the 196 patients with normal chromosome G-binding results showed the following characteristics: the frequencies of ASXL1, DNMT3A and TET2 were very close to the frequencies in all 320 cases (12.8% vs. 12.8%, 10.7 vs. 10.6%, and 10.7 vs. 13.4%, respectively), but these normal karyotype patients rarely exhibited mutations in TP53, RUNX1 and U2AF1 (Fig. 3).

### Impact of individual mutations on survival

**Figure 4:**
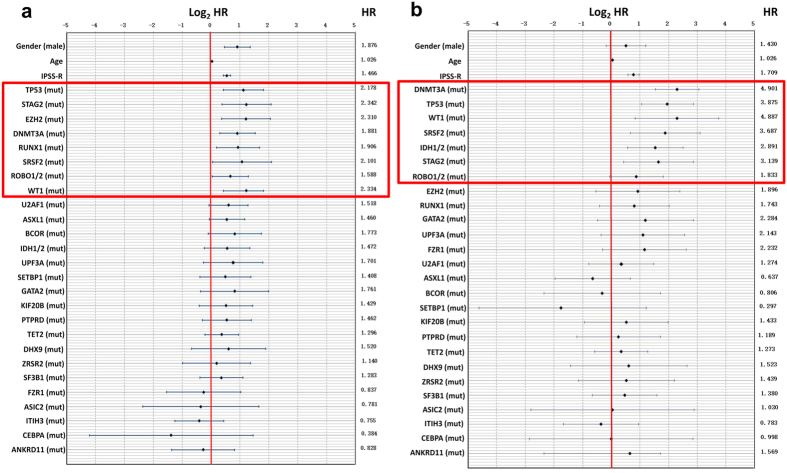
Illustration of the hazard ratios for overall survival (OS) and AML transformation. Hazard ratios (HRs, shown with numbers) as well as logHR values and 95% confidential intervals (shown as a chart) for the parameters used for the OS-predicting system, including clinical and significant genetic mutations (red box, TP53/STAG2/EZH2/DNMT3A/RUNX1/SRSF2/ROBO1&2/WT1/U2AF1) (**a**), and for the AML transformation-predicting system, including clinical and significant genetic mutations (red box, DNMT3A/TP53/WT1/SRSF2/IDH1&2/STAG2/ROBO1&2) (**b**).

We performed univariate analyses to evaluate the influence of gene mutations and other common prognostic factors, including age, gender and IPSS-R, on overall survival (OS). The results showed that male gender (*P* = 6.8E-05, HR = 1.876), advanced age (*P* = 4.9E-08, HR = 1.026), a high IPSS-R score (*P* = 1.0E-13, HR = 1.466) and the occurrence of multiple gene mutations, including TP53 (*P* = 1.0E-03, HR = 2.178), STAG2 (*P* = 5.0E-03, HR = 2.342), DNMT3A (*P* = 4.0E-03, HR = 1.881), EZH2 (*P* = 5.0E-03, HR = 2.310), RUNX1 (*P* = 1.5E-02, HR = 1.906), ROBO1/2 (*P* = 3.6E-02, HR = 1.588), SRSF2 (*P* = 4.1E-02, HR = 2.101) and WT1 (*P* = 4.2E-02, HR = 2.334) mutations, were associated with significantly reduced OS (Fig. 4a). These mutated genes associated with an adverse prognosis were also identified in previous studies^4^,^7^,^8^,^12^. Further multivariate analyses were performed to screen independent prognostic factors. Male gender (*P* = 2.0E-02, HR = 1.503), advanced age (*P* = 4.0E-03, HR = 1.016), a high IPSS-R score (*P* = 4.2E-13, HR = 1.436), WT1 mutation (*P* = 4.5E-02, HR = 2.700) and PTPRD mutation (*P* = 4.8E-02, HR = 1.881) were defined as independent prognostic indicators.

### Impact of individual mutations on AML transformation

Regarding AML-free survival, univariate analyses showed that advanced age (*P* = 1.0E-03, HR = 1.026), a high IPSS-R score (*P* = 1.0E-13, HR = 1.709) and mutation of the DNMT3A (*P* = 3.1E-09, HR = 4.901), TP53 (*P* = 2.5E-05, HR = 3.875), WT1 (*P* = 2.0E-03, HR = 4.887), SRSF2 (*P* = 2.0E-03, HR = 3.687), IDH1/2 (*P* = 2.0E-03, HR = 2.891), STAG2 (*P* = 8.0E-03, HR = 3.139) and ROBO1/2 (*P* = 4.7E-02, HR = 1.833) genes predicted a high AML transformation rate (Fig. 4b). Further multivariate analyses were performed to screen certain independent AML transformation-associated factors. IDH1/2 (*P* = 9.9E-06, HR = 13.394), a high IPSS-R score (*P* = 2.7E-11, HR = 1.729), DNMT3A (*P* = 1.0E-03, HR = 3.105), TP53 (*P = *5.0E-03, HR = 2.966), SRSF2 (*P* = 8.0E-03, HR = 4.664), WT1 (*P* = 9.0E-03, HR = 6.128) and STAG2 (*P* = 3.1E-02, HR = 3.948) were defined as independent AML transformation-associated indicators.

### Novel prognostic model including molecular markers

**Figure 5:**
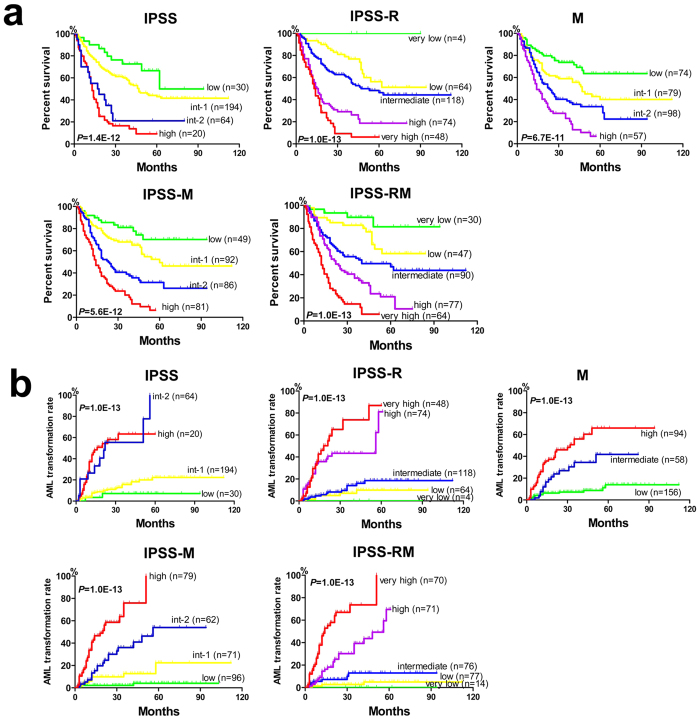
Construction of novel prognostic evaluation systems for OS and AML transformation. (**a**) Kaplan-Meier survival analysis is shown for four groups (low, int-1, int-2 and high) according to IPSS, five groups (very low, low, int, high and very high) according to IPSS-R and four groups (low, int-1, int-2 and high) according to the mutation-based system (in order from left to right). After the integration of IPSS or IPSS-R with the mutation-based system, an improved Kaplan-Meier survival analysis is observed according to IPSS-M and IPSS-RM. IPSS-M, IPSS plus mutations; IPSS-RM, IPSS-R plus mutations. (**b**) Similarly, Kaplan–Meier estimates of AML transformation are shown for four groups according to IPSS, five groups according to IPSS-R and three groups (low, int and high) according to the mutation-based system (in order from left to right). After the integration of IPSS or IPSS-R with the mutation-based system, an optimized AML transformation analysis is observed according to IPSS-M and IPSS-RM.

Based on the significant impact of gene mutations on OS, we constructed a molecular marker-based model for risk stratification using the following definitions: Low: no mutations; Intermediate-1: presence of 1 common mutation (mutations in all genes except for TP53, STAG2, DNMT3A, EZH2, RUNX1, ROBO1/2, SRSF2 and WT1); Intermediate-2: presence of 2–3 common mutations or 1 poor mutation (TP53, STAG2, DNMT3A, EZH2, RUNX1, ROBO1/2, SRSF2 and WT1) plus 0–2 common mutations; High: presence of ≥ 4 mutations (common or poor mutations) or two poor mutations (Supplemental Table 2). Using this system, the 320 patients could be significantly divided into 4 risk groups (*P* = 6.7E-11). To further improve the prognostic stratification, we integrated this mutation-based system into the IPSS or IPSS-R scoring system (Supplemental Tables 3 and 4). The results suggested that whether the four subgroups of IPSS or five subgroups of IPSS-R were employed, the addition of mutation scoring significantly separated the survival curves (*P* = 5.6E-12; *P* = 1.0E-13) (Fig. 5a).

Similar to the OS analysis, we constructed a predictive model for AML transformation based on the significant gene mutations associated with AML transformation using the following definitions: Low: 0–1 common mutations (mutations in all genes except for DNMT3A, TP53, WT1, SRSF2, IDH1/2, STAG2 and ROBO1/2); Intermediate: 1 driving mutation (DNMT3A, TP53, WT1, SRSF2, IDH1/2, STAG2 and ROBO1/2) or 2 common mutations; High: ≥2 driving mutations or the presence of ≥3 common mutations (Supplemental Table 5). The 320 patients could be significantly divided into 3 risk groups according to this system, based on which AML transformation risk was well distinguished (*P* = 1.0E-13) (Fig. 5b). We integrated this mutation-based model into IPSS and IPSS-R (Supplemental Tables 6 and 7), and the results suggested that the IPSS-M or IPSS-MR system achieved better risk stratification for AML transformation than the primary system (*P* = 1.0E-13; *P* = 1.0E-13) (Fig. 5b).

## Discussion

Several next-generation target-sequencing studies on MDS have been reported thus far^7^,^8^,^9^,^10^. However, there are no available data from multiple-target gene-sequencing based on a large Chinese population. As we described in INTRODUCTION, the detectable coverage varies according to the number of target genes^7^,^8^,^16^,^17^,^18^. Additionally, the incidence of MPN-related (JAK2, CALR and MPL) and *de novo* AML-related (N-RAS, K-RAS and FLT-3) gene mutations is low in typical MDS subsets^7^,^8^,^19^,^20^. In this study, 18 genes showing a high frequency of mutation in MDS were adopted for sequencing analysis, including the epigenetic modifiers ASXL1, DNMT3A, EZH2, TET2, IDH1/2 and BCOR; the RNA spliceosome genes SF3B1, SRSF2, U2AF1, and ZRSR2; the transcription factors CEBPA, GATA2, RUNX1, and SETBP1; the adhesion molecule STAG2; and the tumor suppressors WT1 and TP53. Additionally, 10 novel recurrently mutated genes (which showed a higher incidence in MDS in our previous sequencing work and have been reported to be related to carcinogenesis) were employed to obtain a small group of 28 target genes. The ten novel genes were ROBO1/2, ITIH3, PTPRD, KIF20b, ANKRD11, UPF3A, DHX9, ASIC2 and FZR1^12^. Compared with the results reported by Papaemmanuil *et al*. (74% of gene mutations detected in 738 patients using 111 target genes panel) and reported by Haferlach *et al*. (89.5% of gene mutations detected in 944 patients using 104 target genes panel)^7^,^8^, this novel combination of genes allowed the detection of 77.2% of mutation events and 82.8% of genetic events when employed together with karyotype analysis. Thus, we considered this combination to be effective in the detection of gene mutations in MDS. However, this design needs to be further validated with larger sample sizes, and/or performed in other MDS centers to determine its efficiency.

Previous studies have found specific cooperative or exclusive relationships among specific gene mutations^7^,^8^. Our results did not exhibit absolute consistency with previous reports, possibly due to the different races, disease subsets, and choice of target genes involved^7^,^8^. Because of the especially low incidence of SRSF2 mutations in Chinese MDS cases (3.4% vs. 13–15% in foreign literature)^7^,^8^,^20^, the cooperative relationship between the mutations in SRSF2 and STAG2/RUNX1/ASXL1/IDH2 confirmed in the published literature was not validated by our results^7^,^8^. However, we obtained consistent findings with Papaemmanuil *et al*.^7^ and Haferlach *et al*.^8^ regarding the cooperation between EZH2 and ASXL1/RUNX1. Additionally, the exclusive relationship between spliceosomes was explicitly validated^7^,^8^. Interestingly, TP53 mutations rarely co-occurred with RUNX1 or SETBP1 mutations in the same individuals (exclusive *q* value of 0.001). Furthermore, RUNX1 and SETBP1 were rarely mutated together. All three gene mutations have been considered to be closely related to MDS progression^4^,^21^,^22^,^23^. It has been proposed that different signaling pathways may participate in the development MDS in individual cases, and a key mutation may be sufficient for the disease to undergo transformation. Thus, MDS progression could occur through a heterogeneous pathway, unlike a homogeneous model (controlled by signal-transducing and transcription factors) as observed for *de novo* AML.

A comprehensive analysis was carried out between chromosome karyotypes and gene mutations. First, 30 patients with trisomy 8 presented a higher incidence of U2AF1 or ZRSR2 mutations (20% and 16.7%, respectively). However, the “good” karyotype 20q- showed a marked cooperative relationship with WT1, SRSF2 and U2AF1 mutations, all of which are related to poor outcomes according to the literature and our results^7^,^24^,^25^. Patients with −7 or 7q- showed a very close cooperative relationship with RUNX1/SETBP1 (frequencies reaching 50% and 37.5%, respectively), which is highly consistent with the poor outcome for this subset of MDS. However, 5q- MDS patients tended to harbor TP53 mutations, consistent with the findings of Papaemmanuil E^7^. Patients with complex karyotypes in this study showed a highly cooperative relationship with TP53 mutations (also consistent with Papaemmanuil E^**7**^) (incidence as high as 36%), and the inferior personal predictive effects of TP53 mutations and complex chromosomes were highly consistent^26^,^27^. Notably, two of 3 patients with the “very good” (according to IPSS-R) 11q- presented ROBO1 mutations. Mutation detection approached nearly 75% in the 196 patients with normal karyotypes. Two typical MDS-related mutations showed nearly equal frequencies in normal karyotype patients and patients with abnormal chromosomes, in the genes ASXL1 (12.8% vs. 12.8%) and DNMT3A (10.7% vs. 10.6%). Both mutations predicted a poor outcome according to the previous literature^28^,^29^, suggesting that normal karyotypes do not always point to a good prognosis, although we do not consider the outcomes of these chromosomally normal patients to be the same as those of high-risk patients because they rarely display mutations in genes such as TP53, RUNX1 or U2AF1 that have been confirmed to predict an undesirable outcome. These chromosome-related mutation analyses provide new insights.

IPSS or IPSS-R based on prognostic factors including bone marrow blasts, cytogenetics and cytopenias (hemoglobin, platelets and ANC) is considered the most preferred prognostic system^3^,^11^. However, IPSS or IPSS-R does not include gene mutations that have been shown to be associated with disease prognosis in recent studies^4^,^7^,^8^. In studies by our group and others, certain gene mutations have been shown to coexist with specific chromosomal abnormalities^8^,^16^, though this does not mean that they can be equated, considering, for example, that 74.2% (142/192) of MDS patients with a normal karyotype exhibit at least one gene mutation. In addition, IPSS or IPSS-R analysis did not distinguish the survival curves for each group well in the present study, especially among lower-risk groups or higher-risk groups. Therefore, it is necessary to integrate gene mutations into the prognostic system. Mutation-based survival analyses showed that the combination of a group of significant high-risk markers (TP53, STAG2, DNMT3A, EZH2, RUNX1, ROBO1/2, SRSF2 and WT1) and the cumulative effects of mutations in 19 other genes predicted four subgroups with significantly different survival. Therefore, we established a new comprehensive IPSS-M or IPSS-RM system through integrating clinical and mutation parameters, which appeared to generate perfect stratification curves. Similarly, gene mutations were also included in a new AML-predicting model. The combination of a group of significant risk markers of AML transformation (DNMT3A, TP53, WT1, SRSF2, IDH1/2, STAG2 and ROBO1/2) and the cumulative effects of mutations in 19 other genes predicted three subgroups with significantly different AML transformation rates. The IPSS-M and IPSS-RM based on this model generated better-separated curves for leukemia-free survival than the primary system. Similar prognostic models were also reported in previous studies^8^,^30^. In a recent study, Nassau A *et al*. established and validated a predictive model in treated MDS patients through incorporation of 62 mutated genes into IPSS-R. Integrated model also significantly improved the prognostic evaluation of MDS patients regardless of their initial or subsequent therapies. Specifically, EZH2 and TP53 were considered as independent factors predicting poor survival, which is consistent with our findings^31^. Further validating work on our risk model including more newly diagnosed MDS patients (over 300 cases) is being carried out. Validating model will be built according to the calculation mode from the training model. The concordance index will be used to compare the statistical power of the training with validating model. Based on the integration of mutations information from training cohort with validating cohort, independently prognostic factors would be screened to supplement into IPSS or IPSS-R, and improve their prognosis evaluation. In brief, the aim of construction of prognostic models is to supply better prognostic prediction, therapeutic interventions and our knowledge of MDS pathogenesis.

In summary, using a sequencing design involving 28 target genes, we were able to detect at least one gene mutation in over 77.2% of MDS patients. This design is simple and effective and can provide us with valuable information on the pathogenesis and prognostic evaluation of MDS as well as clinical interventions for MDS.

## Additional Information

**How to cite this article**: Xu, F. *et al*. Exploration of the role of gene mutations in myelodysplastic syndromes through a sequencing design involving a small number of target genes. *Sci. Rep.* **7**, 43113; doi: 10.1038/srep43113 (2017).

**Publisher's note:** Springer Nature remains neutral with regard to jurisdictional claims in published maps and institutional affiliations.

## Supplementary Material

Supplementary Tables

## Figures and Tables

**Table 1:** Characteristics of the total cohort.

Parameter	Total cohort (n = 320) patient numbers ratios, ranges or percentages
Males: females (*ratio*)	178:142 (1.25)
Median age (years) (range)	57 (11–91)
MDS subtypes	
MDS-RS	23 (7.2%)
RCUD (RA/RN/RT)	22 (6.9%)
RCMD	156 (48.8%)
RAEB1	69 (21.6%)
RAEB2	49 (15.3%)
MDS with isolated 5q-deletion	1 (0.31%)
Cytogenetics	
Normal	196 (61.2%)
Abnormal	124 (38.8)
IPSS risk group	
Low	31 (9.7%)
Intermediate- 1	201 (62.8%)
Intermediate- 2	68 (21.2%)
High	20 (6.3%)
IPSS-R risk group	
Very low	4 (1.3%)
Low	63 (19.7%)
Intermediate	127 (39.6%)
High	78 (24.4%)
Very high	48 (15.0%)

Abbreviations: IPSS, International Prognostic Scoring System; MDS, myelodysplastic syndrome; RCUD, refractory cytopenia with unilineage dysplasia; RAS, refractory anemia with ringed sideroblasts; RCMD, refractory cytopenias with multilineage dysplasia; RAEB, refractory anemia with excess blasts.
