# Supplementary material for: Exploration of the role of gene mutations in myelodysplastic syndromes through a sequencing design involving a small number of target genes
Source: Sci Rep. 2017 Feb 21;7:43113. doi: 10.1038/srep43113 (PMC5318910; doi:10.1038/srep43113)
Supplement: Supplementary Tables [file srep43113-s1.doc]

**Exploration of the role of gene mutations in myelodysplastic syndromes through a sequencing design involving a small number of target genes**

Feng Xu^1,2^, Ling-Yun Wu^1,2^, Qi He^1^, Dong Wu^1^, Zheng Zhang^1^, Lu-Xi Song^1^, You-Shan Zhao^1^, Ji-Ying Su^1^, Li-Yu Zhou^1^, Juan Guo^1^, Chun-Kang Chang^1,*^, Xiao Li^1,*^

^1^Department of Hematology, Shanghai Jiao Tong University Affiliated Sixth People's Hospital

^2^Feng Xu and Ling-Yun Wu have equal contribution to this work.

*Correspondence to: Xiao Li, MD, PhD, [lixiao3326@163.com](mailto:lixiao3326@163.com); Chun-Kang Chang, MD, [changchunkang7010@aliyun.com](mailto:Changchunkang7010@aliyun.com). Dept. of Hematology, Shanghai Jiao Tong University Affiliated Sixth People’s Hospital, Shanghai, China, 200233;

**Supplementary Table 1. Corresponding mutations by some special abnormal chromosome**

|  | **Total**  （320） | **Normal**  （196） | **+8** (n=30) | **20q-**  (n=13) | **5q-**  (n=7) | **7 involve**  (n=8) | **11q-**  (n=3) | **1p-**  (n=5) | **Complex**  (n=36) |
| --- | --- | --- | --- | --- | --- | --- | --- | --- | --- |
| ***ANKRD11*** | 4.4 | 2.6 | 6.7 | 15.4 | ─ | 12.5 | ─ | 20.0 | 8.3 |
| ***ASIC2*** | *1.25* | 2.0 | ─ | ─ | ─ | ─ | ─ | ─ | ─ |
| ***ASXL1*** | *12.8* | 12.8 | *22.6* | 15.4 | 14.3 | 12.5 | ─ | 20.0 | 8.3 |
| ***BCOR*** | 4.4 | 5.1 | 6.7 | ─ | ─ | ─ | ─ | ─ | 5.6 |
| ***CEBPA*** | *0.94* | 1.5 | *─* | *─* | ─ | ─ | ─ | ─ | ─ |
| ***DHX9*** | 2.5 | 3.6 | 3.3 | ─ | ─ | ─ | ─ | ─ | 2.8 |
| ***DNMT3A*** | 10.6 | 10.7 | 3.3 | 7.7 | ─ | 12.5 | ─ | ─ | *19.4* |
| ***EZH2*** | 4.1 | 3.1 | 6.7 | ─ | ─ | 12.5 | 33.3 | ─ | 8.3 |
| ***FZR1*** | *2.2* | 3.6 | *─* | *─* | ─ | ─ | ─ | ─ | ─ |
| ***GATA2*** | 2.5 | 3.1 | 3.3 | ─ | ─ | ─ | ─ | ─ | ─ |
| ***IDH1/2*** | 6.9 | 8.7 | ─ | 7.7 | ─ | 25.0 | ─ | ─ | 2.8 |
| ***ITIH3*** | 8.4 | 6.6 | 10.0 | 15.4 | 14.3 | 25.0 | ─ | ***40.0*** | 11.1 |
| ***KIF20B*** | 8.4 | 5.6 | 3.3 | 7.7 | ─ | ─ | ─ | ─ | 2.8 |
| ***PTPRD*** | 5.6 | 6.1 | 6.7 | ─ | ─ | 12.5 | ─ | ─ | 2.8 |
| ***ROBO1/2*** | 10.9 | 8.7 | 16.7 | 15.4 | 14.3 | ─ | ***66.7*** | ─ | 13.9 |
| ***RUNX1*** | 7.5 | 2.6 | 10.0 | 7.7 | ─ | ***50.0*** | ─ | 20.0 | 13.9 |
| ***SETBP1*** | 4.7 | 3.1 | 3.3 | ─ | ─ | ***37.5*** | **33.3** | ─ | 2.8 |
| ***SF3B1*** | 7.5 | 7.7 | 3.3 | 15.4 | ***28.6*** | ─ | 33.3 | ─ | 2.8 |
| ***SRSF2*** | 3.4 | 3.1 | ─ | ***23.1*** | ─ | ─ | ─ | ***20.0*** | 5.6 |
| ***STAG2*** | 4.4 | 5.1 | 6.7 | ─ | ─ | ─ | ─ | ─ | 2.8 |
| ***TET2*** | 13.4 | 10.7 | 20.0 | ─ | ─ | 12.5 | ─ | 40.0 | 16.7 |
| ***TP53*** | 7.2 | 0.5 | 3.3 | 7.7 | ***28.6*** | ─ | 33.3 | ─ | ***36.1*** |
| ***U2AF1*** | *9.4* | 4.6 | ***20.0*** | ***38.5*** | ─ | ─ | ─ | 20.0 | 8.3 |
| ***UPF3A*** | 3.4 | 3.6 | ─ | ─ | ─ | ─ | **33.3** | ─ | 5.6 |
| ***WT1*** | 1.6 | 0.5 | ─ | **15.4** | ─ | ─ | ─ | ─ | 2.7 |
| ***ZRSR2*** | 2.8 | 1.5 | **16.7** | ─ | ─ | ─ | ─ | ─ | ─ |

**Note：**1. Aside from Complex, all chromosome abnormalities means sole or at most one additional abnormality; 2. bold and italic fonts means mutations presenting statistically cooperative relationship with that special chromosome abnormalities.

**Supplementary Table 2 Mutation-based prognosis system for OS**

| Prognostic variable | Low risk | Int-1 risk | Int-2 risk | High risk |
| --- | --- | --- | --- | --- |
| *Mutation index | 0 | 1 | 2-3 | ≧4 |
| Scoring description | No mutation | 1  ^1^general mutation | 2-3 general mutation or 1 ^2^poor mutation plus 0-1 general mutation | ≧4 general mutations or ≧2 poor mutations or 1 poor mutation plus 2-3 general mutations |

^1^General mutations including ASXL1, U2AF1, SETBP1, IDH1/2, BCOR, UPF3A, KIF20B, PTPRD, GATA2, TET2, DHX9, ZRSR2, SF3B1, FZR1, ASIC2, ITIH3, CEBPA, ANKRD11 mutations;

^2^Poor mutations including DNMT3A, SRSF2, STAG2, TP53, EZH2, RUNX1, ROBO1/2, WT1

*Mutation index was defined as follows: each general mutation was scored 1, and each poor mutation was scored 2. Total scoring, also called mutation index, was required by adding these two together.

**Supplementary Table 3 IPSS-M** prognosis scoring system

| Prognostic variable | 0 | 0.5 | 1 | 1.5 | 2 |
| --- | --- | --- | --- | --- | --- |
| Marrow blasts (%) | <5 | 5-10 | --- | 11-20 | 21-30 |
| Karyotype | good | intermediate | poor | --- | --- |
| cytopenia | 0-1 | 2-3 | --- | --- | --- |
| Mutation index | 0 |  | 1 | 2-3 | ≧4 |

**Supplementary Table 4 IPSS-RM** prognosis scoring system

| Prognostic variable | 0 | 0.5 | 1 | 1.5 | 2 | 3 | 4 |
| --- | --- | --- | --- | --- | --- | --- | --- |
| Cytogenetics | Very Good | - | Good | - | Interme-  diate | Poor | Very Poor |
| Bone marrow blast (%) | 2 | - | > 2 - <5 | - | 5-10 | > 10 | - |
| Hemoglobin (g/dL) | 10 | - | 8- <10 | < 8 | - | - | - |
| Platelets (×109/L) | 100 | 50 -< 100 | < 50 | - | - | - | - |
| ANC (×109/L) | 0.8 | < 0.8 | - | - | - | - | - |
| Mutation index | 0 |  | 1 |  | 2-3 |  | ≧4 |

**Supplementary Table 5 Mutation-based prognosis system for AML transformation**

| Prognostic variable | Low risk | Intermediate risk | High risk |
| --- | --- | --- | --- |
| Mutation index | 0-1 | 2 | ≧3 |
| Scoring description | 0-1 general mutation | 2 general mutations or 1 driving mutation | ≧3 general mutations or ≧2 driving mutation |

^1^General mutations including ASXL1, U2AF1, SETBP1, EZH2, RUNX1, BCOR, UPF3A, KIF20B, PTPRD, GATA2, TET2, DHX9, ZRSR2, SF3B1, FZR1, ASIC2, ITIH3, CEBPA, ANKRD11 mutations;

^2^Driving mutations including DNMT3A, WT1, TP53, SRSF2, STAG2, ROBO1/2, IDH1/2

*Mutation index was defined as follows: each general mutation was scored 1, and each driving mutation was scored 2. Total scoring, also called mutation index, was required by adding these two together.

**Supplementary Table 6 IPSS-M-AML predicting scoring system**

| Prognostic variable | 0 | 0.5 | 1 | 1.5 | 2 |
| --- | --- | --- | --- | --- | --- |
| Marrow blasts (%) | <5 | 5-10 | --- | 11-20 | 21-30 |
| Karyotype | good | intermediate | poor | --- | --- |
| cytopenia | 0-1 | 2-3 | --- | --- | --- |
| Mutation index | 0-1 |  | 2 |  | ≧3 |

**Supplementary Table 7 IPSS-RM-AML** predicting scoring system

| Prognostic variable | 0 | 0.5 | 1 | 1.5 | 2 | 3 | 4 |
| --- | --- | --- | --- | --- | --- | --- | --- |
| Cytogenetics | Very Good | - | Good | - | Interme-  diate | Poor | Very Poor |
| Bone marrow blast (%) | 2 | - | > 2 - <5 | - | 5-10 | > 10 | - |
| Hemoglobin (g/dL) | 10 | - | 8- <10 | < 8 | - | - | - |
| Platelets (×109/L) | 100 | 50 -< 100 | < 50 | - | - | - | - |
| ANC (×109/L) | 0.8 | < 0.8 | - | - | - | - | - |
| Mutation index | 0 |  | 1 |  | 2 | 3 | ≧4 |
